# Proceedings of the Southern Dental Association, Conjointly with the North Carolina State Dental Association.—Thirteenth Annual Meeting

**Published:** 1881-10

**Authors:** 


AMERICAN JOURNAL

OF

DENTAL SCIENCE.

Vol. XV. THIRD SERIES—OCTOBER, 1881. No. 6.

### *Southern Dental Association.—Annual Meeting.*

(CONJOINTLY WITH THE NORTH CAROLINA STATE DENTAL ASSOCIATION.)

The thirteenth annual meeting of the Southern Dental Association, conjointly with the North Carolina State Dental Association, was held at Ashville, North Carolina, commencing July 26th, 1881; Dr. V. E. Turner, of Raleigh, N. C., presiding. After prayer by Rev. D. H. Buell, Dr. J. T. Griffith, of North Carolina, delivered a brief and excellent address, extending a cordial welcome from the North Carolina State Dental Association to the Southern Dental Association: "We are proud to say that our State Association, though young, is in excellent working condition, and fully imbued with the spirit of the times. Our membership is steadily increasing, our annual meetings well attended; there is every indication of increased zeal, and a strong disposition on the part of the brethren to be "instant in season and out of season," never forgetting to maintain our integrity as professional men and brethren. The great distance to be traversed prevents the attendance of many. In their absence I stand before you as their representative, and express the sentiment of every reputable dentist in this commonwealth. Honored as we have been in the merited promotion of one of our number to the presidency of your Association; proud as we are of its history and its great work in the elevation of Dentistry to an acknowledged, honoured and legally recognized place among the liberal professions, we esteem it no small honor that you have at length heeded our oft repeated invitations to hold your sessions in our midst. We welcome you cordially. Together in suffering, in mourning over our dead, together in humiliation, poverty and self-denial, so together have we risen superior to every discouragement to-day, until *nowhere* does our chosen profession occupy a higher or more justly won position, than in this regenerated South. Of kindred blood, of kindred sympathies, of kindred aims, we bid you *thrice* welcome to North Carolina; welcome to the hospitalities and health giving breezes of this beautiful queen city of our lovely 'land of the sky.' Welcome to our grand mountains and our beautiful waters. May your stay among us be pleasant, and your memories of this meeting be equally as pleasant in their personal and social reminiscences, as in their recollections of professional improvement."

Dr. George H. Winkler, of Georgia, responded in happy terms, accepting the hospitality so generously offered, which was indeed appreciated.

Dr. Winkler related in touching terms an incident occuring in the dark days of '65, when he, a ragged boy, returning from the wars, was kindly received by a North Carolina farmer; and this present welcome was evidence that the spirit of hospitality was still in the hearts of the sons of the "old North State."

"The objects of our Association are so well known and appreciated that I need not reiterate them here. Our hopes for the future as represented by what we have accomplished in the past, are bright. We are steadily advancing, and in the near future I see one cherished calling occupying the best and most dignified plane it can ever attain. One potent factor in its advancement, peculiar, the characteristic and grand feature of our profession, is the harmonizing within itself of these two branches of secular education, the Physical and Mental. In the learned professions the *mind* is developed and educated, frequently not only without any corresponding development of the physical nature, sometimes even to its positive detriment; while trades and mechanics are conducted by workmen, who are almost devoid of mental attainments. The natural result of this is that labor is degraded, and the artisan meets with social ostracism; while the learned professions are filled with men, who, perhaps fearing this degradation, crowd into them with few of the prerequisite qualifications.

Not so, Mr. President, with our noble calling. The skilled Dentist, representing the harmonious blending of mental and physical education, ennobles labor by his intelligence, and performs his work with practised hands directed by an educated mind; and fair science herself lights him on his way, while sturdy art accompanies him in the completion of his perfect work.

Let us venture the hope that the work accomplished at this session of the Southern Dental Association may go down to the future, rendering our meeting memorial in the annals of Dental Science. Let us hope that such new modes and methods may be proposed, such new principles proclaimed and endorsed, as shall bring the hearty commendation of our brethren every where. Let us hope that some new truth may be enunciated, which like a beacon light shining forth from the heights, shall rejoice the the hearts of all who see it, and guide them upon their way in a still more radiant future.

Let us then one and all strive to establish some fact, however humble it may be, or arrive at some new truth. We cannot estimate the vast importance that may attach to what may seem to us, a minor truth; for who can justly estimate the importance of any point of truth, abstracted from the rest. No matter how meagre its intrinsic worth may seem, it may be an essential factor in some problem of vast application, and its discovery should not be despised : should we fail in our search, we still realize that while

> "Glorious it is to wear the crown,
> Of a deserved and pure success;
> He, who knows how to fail—has won,
> A crown whose lustre is not less."

We cannot fail of an object, unless we have striven for it, and we should remember that whether searching after some humble fact, or reaching after some stupendous truth, there is no science that can inspire one heart with greater zeal, or fill our souls with more lofty enthusiasm than that in whose interests we are gathered to-day.

You have assured us of your friendship, and let us assure you of our enduring love and esteem. We are glad to meet you, young men, upon whom must rest the responsibility of advancing your profession, for we feel that if your faces are an index to your minds, the responsibility has been well placed upon those whose souls are filled with enthusiasm, and whose hearts throb with devotion.

And to you, gentlemen, whose whitening heads bespeak the heat and burden of the day well borne, we look to-day in earnest veneration, as the tried and faithful followers of a beneficent calling, which has 'inspired you, with a desire to dedicate your days to the good of your race, so that in the fading light of life's evening, on looking back you indeed can not be forced to acknowledge, that useless and unsubstantial were the objects *you* pursued.'"

The annual address of the President, Dr. V. E. Turner, of North Carolina was then delivered, in which after modestly disclaiming fitness for the position to which he had been elected, tendering thanks for the honor, and begging forbearance for shortcomings, he said :

"As you all doubtless felt and know our last meeting in New York City was an unusual one in many respects, and we hoped it might do much good towards establishing a truly national body of dentists. How far the movement was successful remains to be seen. We are conscious of having done our whole duty, solely without personal ambition. Our whole motive was the desire to assist in laying the ground-work of an organization which should take its place in the circle of the sciences, and reveal to future generations that we were not unmindful of the duty we owe to them.

Those of us who were present were compelled to confess to the impression of an apparent want of zeal on the part of many of our northern brethren, to say nothing of some exhibitions of opposition. Still it is not for us to censure or condemn the actions of those who differ from us in the manner of promoting and fostering the interests of a cause dear to us all. We must leave it to their own consciences to do their duty in their own way. But we all came back with the firm conslusion that we were done with this coquetting by the other associations; that hereafter we would do our utmost to hold up and strengthen this Southern Association, for it is not without a history of its own, reaching back through a period of over twelve years and embodying much that would reflect honor upon any organization.

Among its members are many honored names, whose genius and culture have produced papers of the highest order and brought to light many facts which are now relied upon as the best authority among the teachers of our science. One especially, who once presided over this body, gave to the world one of the most important discoveries ever made in connection with Dental Science, and which has created an entire revolution in the principle and manner of applying gold to diseased or decayed teeth, and to-day there are thousands of our best men, blessing the name of Arthur for enabling them to make gold cohesive. And I recall the memory of the genial and chivalrous Mc Quillen, whose sparkling mind so often gave interesting and important instruction in our halls; and who will never be forgotten by those who admire unselfish devotion to the cause of science. Both of these gentle spirits, though gone to their reward, are with us still in spirit as we daily discharge our duties, and are conspicuous in the history of Dental Surgery ; and there are many others still battling in the cause, whose names have become famous in the annals of science.

Our society has brought under its influence many unambitious men, who had no conception of their responsibility, and had no desire to understand the principles which should guide their diagnosis and treatment ; and many with no higher motive than to escape more unpleasant labor. We have held out a friendly hand to this class of men and many have been aroused to higher aspirations, and are becoming more worthy of their calling.

Our desire to perpetuate and build up this organization is not tinged with the slightest antagonism to the new society, the National Dental Association, for many of our members belong to it and are working to sustain it. We need this Southern Association for our own convenience—the new one, according to its adopted laws, will meet in the South only at long intervals, and those of us who can not follow it around feel the necessity of an association which shall meet every year within our own borders and among our own people.

With these allusions to the events of the last year I must now, in compliance with a long established custom, refer briefly to the work of Dental Associations.

The history of our specialty has so often and appropriately been the theme of speakers and writers at our annual meetings, and has become so familiar to all that I do not propose to review all the stages of progress which bring us to Modern Dentistry. Whatever has been accomplished has been done within our own ranks, and is due to no outside helping hand, not even from those so closely allied to us. Let it suffice to say that a small band of philanthropists spent years in culling the fragments of science, scattered here, and from distant fields, bringing together the results, and deducting laws for practice by which some of the problems of nature could be solved, thus promoting the health and happiness of their fellow creatures. As the light began to dawn upon these few faithful, earnest men, serious attention was turned in that direction and gradually men of more genius and cultivation were ranked among the students of Dental Surgery, who year by year extended its usefulness and influence, and to-day we find it represented in almost every civilized land by men of skill and culture. The days when only the rich employed the dentist have passed away, and the dentist of to-day is a household necessity, not only to relieve suffering but to promote health and happiness, to say nothing of adding beauty and sweetness to the human face. This position of usefulness has been attained by dint of hard labor and study. And now having stood alone during the dark period of our infancy, we need no patronage or support which we cannot claim in our own right. But those restless and unsatisfied spirits who have been so long possessed with a mania for *recognition*, who have been pining for entrance into the æsculapian temple, have at last found a realization of their hopes, for at the International Medical Congress soon to convene in London, there is provided a special section for the discussion of Dental Surgery by dentists and to which meeting many dentists from this country and some from this Society are now crossing the ocean. Although there has been no doubt of this fact in the minds of the best authority, still as we grew up independently and not as an offspring, like the other specialties, until now, there has been wanting the formality of a full and open recognition. This matter is now fully settled by the decision of a body composed of representative medical men from all parts of the civilized world.

So far as I know there has been within the last year no startling discoveries or conspicuous strides in the matter of progress, nor any special development in practice.

It gives me much pleasure to offer my congratulations to the profession upon the exit of the Goodyear Dental

Vulcanite Company. There are few dentists in the land who do not feel a sense of relief that the marauding agents of that exacting corporation shall no more darken our doors, or dog our footsteps. Not that I value the material or process so highly as a base for artificial teeth, still it is a pleasant change to be able to use it or not without paying an unjust and high royalty.

I am much gratified to observe an unusual interest directed to utilizing many old roots or fangs of natural teeth, which often remain in the mouth quite comfortable for many years after the crowns have been lost. Dr. Bonwill and others have invented some new methods of pivoting of great value, as obviating the great difficulty hitherto felt of preventing access of fluids into the junction of the crown with the root. Thus one serious objection to artificial crowns has been overcome, and by this means many mouths can be treated without the application of plates, which are always productive of injury to the remaining natural teeth. Many teeth can thus be usefully allowed to remain in the mouth which are otherwise often sacrifical. Artificial teeth on plates, no matter how perfectly manufactured or accurately adjusted, are at best but poor substitutes for natural organs, and it behooves us to avoid such a dire necessity whenever possible, hence these efforts to build up or add crowns to all healthy roots are steps in the right direction and one worthy of your serious consideration.

There seems to be an increasing interest directed to the diseases of the gums, more especially that which is known as 'Riggs disease.' It is almost impossible to over estimate the importance of this long neglected duty. Decay is not more fatal and often not so rapid. In many sections of the country it is an alarming evil, which threatens not only to destroy all of the teeth, but produce such a shrinkage of the alveolar ridge that artificial teeth are worn with great difficulty. In many families it seems to be hereditary, and if not looked after at an early age soon reaches a point beyond which treatment is very difficult if not unavailing. I am gratified to notice that some members of our profession are making a specialty of the treatment of these wasted tissues, and would suggest that this body might find it advantageous to appoint a special committee every year, whose duty it shall be to inaugurate some national plan of investigation, and report what progress has been made in the treatment and prevention of this disease.

The fight between the soft and cohesive gold advocates goes on with unabated warmth and ardor. Neither party has been able to give the other anything conclusive as to his own better way of accomplishing the same result. Among the soft foil advocates, there is a strong conviction that to use exclusively cohesive gold and the mallet, involves the necessity of too much loss of the material of the teeth in the preparation of cavities; that teeth are mutilated to facilitate such operations, and that there is a greater danger of leakage. On the contrary the cohesive gold advocates seem unable to believe that soft gold can be made sufficiently dense to answer permanently. These extremes, like radical opinions upon all subjects, are apt to be wrong and dangerous, for they leave the student completely at sea as to which course he ought to pursue. In reality the results are not very different when applied by skillful hands, and at last this is the whole secret. A thorough understanding of the capabilities of either soft or cohesive gold, and a careful manipulation of either, will almost always result in a respectable degree of success, and it should be our ambition to comprehend the peculiarities of both systems of stopping teeth; in fact we should endeavor to be always guided by the indications presented and apply that which our intelligent discrimination directs.

In professional life it is hazardous to become wedded to to any one plan of treatment, regardless of the experience of others. It is not given to any one man, in his brief span, to know all that may be learned concerning these matters of science ; it is by placing our little mites together that a system of results becomes known and is recognized as the best authority, and as such, is handed down to the student of dental science.

In recognizing the great strides made in our profession during the last forty years, we must not forget that in somewhat the same ratio have our difficulties and complications increased, for we cannot fail to see how great has been the deterioration of the structures with which we have to deal. If the well-informed dentist of to-day had the treatment of the Dental development of a century ago, he would find the results very different from his actual experience. Hence it is that many intelligent persons ask, Why is it that with all this boastful progress we find so many more absent teeth and vacant jaws? To meet this increased susceptibility to decay we must bring to the task a higher degree of preparatory education. Our investigations should go beyond our operating room and laboratory. We must take the responsibility of looking far back into the realms of intra-uterine life, to embryology and even farther. We must scrutinize the conduct and health of her who is to be the mother of our future patient, and see that she is properly instructed in those laws of nature which can not be broken without a penalty. We must deal with this delicate subject in such a manner as to convince her of its great importance and of her fearful responsibility. To accomplish this it is not necessary to transcend the bar which custom and our education have thrown around this subject. A general diffusion of such knowledge would soon be effective; for whatever other faults a woman may have, she is prepared at all times unhesitatingly to make any sacrifice for her offspring, and when she knows that health and happiness are involved, you will find her equal to the situation.

How many mothers, except among the most thoroughly educated, understand to what extent their own health and conduct must determine the fate of their children ? How many have any guide except the maternal instinct, which only teaches her to love them with the deepest and most tender affection known to nature? Beyond this they are woefully ignorant of the proper care to be exercised. Is it not a startling fact that so few know how to feed and clothe their children, when this is about all that is required during the first few years of their existence.

Much of this deterioration of teeth is an out-crop of our modern luxurious way of living. There is a lack of that kind of food which is rich in the elements of tooth structure, but this is not the whole truth. We cannot feed the teeth without there is a thoroughly healthy and vigorous development of the other organs. Another great bane of this age is the fearful mistake which is made in endeavoring to educate children too early in the cultivation of the mental powers to the utter neglect of the physical development. There can be no doubt that the Creator intended there should be perfect harmony in the development of the physical and nervous systems, and when such has been preserved we come nearest to a perfect organization. Any mode of life which destroys this harmony will cause deterioration of many if not all the organs, and it is a well-known physiological law that if the brain and nervous system are unusually active, the strain upon the sources of nutrition will be at the expense of the physical system. This is why we so often see in the pale faces of tender, feeble children, bending under a load of books, as they trudge along to school, an anxiety and thoughtfulness far beyond their years. Why is it we must take up these burdens before life is fairly begun? Why shall they not be permitted to grow in peace, like the young of everything else in nature? Whence come these nervous and prematurely old women whose very lives seem to be made up of aches and pains which have no existence in fact, but the influence of which over them (and all of their friends) is even worse then actual physical suffering? Why is it that so many thousand men and women are afflicted with what are called nervous diseases, which were unknown to our great grand-parents? Why are all these perils which beset the modern mother? It is because at that period of infancy, when nature intended our physical natures to be devoloped, these were exhausting their youthful powers in efforts to master some complex science far beyond their years; dwarfing their physical economy to develop a mind, whose powers are destined to be useless on account of a frail body.

Gentlemen, the important work before us demands our best talents, and a combined effort is always more effective and progressive, for nothing so expands the heart and mind and brings the glow of generous emotion like the interchange of fraternal greetings and the free and liberal comparison of ideas. When we measure ourselves by the side of others, by giving expression to our thoughts, they assume more definiteness and clearness, and we feel a greater enthusiasm and a stronger thirst after all that may be known concerning the subject. Dentists like other men, cannot live alone, and to prevent the debasement of our powers, God has surrounded us with mighty and various influences and impulses, which mould our characters and make us useful and wise. We gather new energies from the success of others. We feel inspired by the genial warmth and sympathy which must brighten our minds and give us new strength to battle or to suffer for the accomplishment of good. Without this associated effort we would soon dwindle into selfish views of life and all its duties. The stubborness which ignorance brings would soon warp our judgment, and all chance of progress would be ended. The man who feels that it is not his duty to be identified with these associations, requires but little knowledge of history of human nature to prove that he is not only untrue to himself, but to his profession and to his patrons. Seclusion from those of our own guild brings contracted ideas and a contracted heart; a morbid hatred to light, a cowardly, jealous dislike toward all who are superior. Association brings liberal opinions, a warm, generous heart, a cheerful disposition, a desire for the success and happiness of all, an open admiration for all that is meritorious in others and a brave and chivalrous adherence to truth, let it come when and how it will.

In conclusion, allow me to say that there is something very impressive in this gathering of men, fresh from their fields of labor, from all parts of the country, intent upon discovering the best means to relieve human suffering and endeavoring to contribute to the general fund of scientific knowledge. And it is manifest that our thinking and working men are resolutely coming forward, determined to sacredly hold fast to all that has been gained in the past and to employ their best energies in developing the greatest possibilities of the future."

At the conclusion of the address of the President, Drs. J. T. Griffith and M. A. Bland were appointed to fill two vacancies on the executive committee, and on motion the courtesies of the floor were extended to resident and visiting dentists and physicians of Asheville.

Dr. Walker, of New Orleans, made a few remarks apropos to the occasion. He complimented the eloquent address of Dr. Griffith, and the equally eloquent address of Dr. Winkler, and said that for several years this Association had been engaged in missionary work, in the mountains of Maryland, at the falls of Niagara, and the great city of New York, for the purpose of forming the most perfect, practical and admirable dental association in existence; and he was happy to say that our efforts had been eminently successful, and in the face of uncalled for and unreasonable opposition, we had organized in New York City last year an association which shall be truly national in character, and that will meet consecutively in the various districts of the United States, thus giving all sections an opportunity of enjoying its benefits. He was glad to be again at home, in the mountains of the old North State, and amidst the grand scenery of these mountains. He trusted that these lofty peaks which surrounded us may be typical of the glorious heights of science toward which we shall make a long stride at this meeting, and that the glorious sunshine on their cloud-kissed heights shall be typical of the light of scientific truth here brought out. Adjourned.

---

*Afternoon Session.*

The Association re-assembled at 4 P. M., the President in the chair. The routine business over, the Secretary, Dr. Chisholm stated that the roll needed revision greatly, many whose names were found there, being dead, or had ceased to be members from non-attendance or other causes, the constitution not providing for a revision of the roll by the Secretary, and suggested that a committee be appointed with power to make a complete revision of the roll of membership. After further debate the suggestion was postponed for subsequent action.

The Chairman of the Executive Committee reported the following order of business: Clinics—8.30 A. M. to 10.30 A. M.; Morning Session—10.30 A. M., to 2 P. M.; Afternoon Session—3.30 to 6 P. M.; Night Session—8.30; adopted. After routine business, collection of dues, &c., the Association adjourned to meet at 10.30 A. M., Wednesday.

---

SECOND DAY,
*Morning Session.*

The President in the chair. After the routine business the report of the Committee on Dental Education was read by Dr. George H. Winkler, of Georgia, as follows:

Education, when considered in relation to a special calling in life, may be divided into two distinct stages of progress.

1st., General, or preparatory; 2d, Special, or practical.

1st. Under the head of general education may be considered such acquirement of information as may fit the student for any branch of study, and will not materially differ as a preparation for any one of the learned professions. It is an acknowledged fact that the more complete and thorough this general education may be, the more thoroughly equipped will the student be for beginning the study of his special department, whatever that may be. In this country a thorough knowledge of the English language, some acquaintance with the classics, mathematics and the general principles of natural philosophy and mechanics would scarcely be considered too wide a range for a general or preparatory education prior to beginning the study of any of the special departments of professional life. The, kind and the amount of knowledge above referred to would not fit anyone, the most talented even, to practice any single profession ; but it would be a good preparation for beginning the study of any one of them, whether medicine, theology or law; and further, after acquiring the special knowledge necessary to enter any of the learned professions, he would be prepared to bear himself creditably in his intercourse with his fellows and with the members of other learned professions with whom he might come in contact.

2d. The special or practical education being that required to carry out the aims and ends of a useful life, of course bears more directly upon the department contemplated by the student. In the medical profession as well as the dental, it is well known that of those who have practiced their respective callings creditably and successfully, a large number began study and preparation here, that is with the acquisition of such knowledge as had a direct bearing upon the daily work of life. Yet to present what we regard as the true ideal of a dental education, your committee cannot do less than call attention to the importance of combining in some form or other the two grades of training above referred to, viz., 1st., the *preparatory*, and 2d., the *special instruction*, of those who would pursue the study and practice of our profession most creditably and successfully.

What, then, is the *best* preparatory education for entering the Dental Profession?

Briefly and comprehensively as is possible at this time, yet hesitating on account of the new and, what will be considered by some, questionable ground we are about to enter upon, we answer: Did your committee dare to give without ample argument and full statement their views upon this subject, they would state, (rather as a hope for the near future than as a method for universal adoption just at the present time) that the preliminary professional education of the dental practitioner or dental surgeon after having passed through a liberal academic course, should invariably, or at least whenever possible, include a thorough study and graduation in the general science and profession of *medicine.* Besides the theoretical and practical advantages incident to the knowledge acquired by such a course of preparation, this would place our calling in life upon the best and most dignified plane that it can ever hope to reach. It will be associating ourselves with a noble and venerable profession, and making our department a fully recognized and respected *specialty of that profession.* The surgeon, the obstetrician, as well as the ophthalmologist, the aurist and laryngologist, while following their own special lines of study and making themselves known in their particular departments, are still recognized as brethren in full fellowship with all other physicians. Would it not be, we ask, an object to be desired that at some future time our own cherished calling should become also aligned and fully associated with the medical profession, with which by many affinities and sympathies we are already closely related. In making the proposition your committee is well aware of the many circumstances which at present might appear unfavorable and obstructive to its successful accomplishment. But it will be at the same time recollected that it is not so much for the present, as for a future generation, that we have to realize this ideal of progress. Few of us, perhaps none of us, may live to enjoy the benefits and the increased dignity our profession will attain by the method and the association which your committee here proposes; yet it must be recollected that we do not live altogether in the transient present, but beyond and above our present surroundings we direct our eyes toward a still more elevated and expanding future which our beloved profession will attain and perpetuate, and what we do now to magnify and dignify her will be like the blocks of granite carried generation after generation by the devout and patriotic Egyptians. The pile grew slowly and almost imperceptible while generation after generation of workmen died and were gathered with their fathers to Iris and Osris. But as centuries rolled on and the glory of Egypt became refulgent, the pyramids were completed, and forty centuries have looked down upon their massive structure as one of the architectural wonders and mysteries of the world."

Prof. Winder.—This is a matter of the utmost importance, and I hope this body will move slowly and carefully, looking deeply and considering well before endorsing all that is contained in this report. The line of demarkation must be drawn between Medicine as embracing the whole of the grand art of healing, and "M-D-ism." The science of medicine, or that part of it which I have called "M-D ism" must some day become split up into branches, or specialties such as oculists, etc. Indeed it is practically so at present in most of our cities. I am opposed to merging Dentistry into it. There should be a distinct line between the degrees of M. D., and D. D. S. I have had hopes for some years past that the day would come when there would be Dental Surgeons in the United States Army and Navy, on an equal footing with medical men proper. The officers of each branch of the United States Service advocate this step, and in fact the only opposition comes from the medical men. Think of it, these officers and men, with their families, are often sent to out-posts on the frontier for many years with no one to look after their teeth. If we wish to gain this position, evidently we must hold our position separate and distinct from that of medicine. I am unwilling to place this Association or myself on record in this matter until we are positively sure of what we are about to do, and whither such a step will lead us; and while I have great respect for the M. D., still we must remember that in the infancy of our profession all the medical institutions that were asked to aid in its advancement turned a cold shoulder to us, and we had to struggle on alone. Now that we have progressed so grandly in this noble work, we are competent to take care of ourselves, and I for one, propose that we still hold ourselves as a distinct profession and not as a specialty of "M-D-ism." If you want anything well done, do it yourself. We do not need, nor do we ask, the favor of the medical profession; we ask only justice, and as they say that dentistry is *not* a branch of medicine, we should hesitate before voting ourselves into their ranks.

The Johns Hopkins University, of Baltimore, is the youngest, richest and strongest institution of the kind in the country. The President of that school, Prof. Gilman, places dentistry on exactly the same plane as medicine, and that school will teach Anatomy, Physiology and Chemistry to medical and dental classes (when they have them,) alike. In this respect this school will differ from any other teaching medicine, in having chairs teachhing both medical and dental students these three studies; when these studies are completed, the student passes into the medical class proper or dental as he elects. But he must first graduate in these three studies. Under present circumstances, I cannot think it best for this Association to pledge itself as it would by the adoption of this report.

Dr. G. H. Winkler.—I think my friend, Dr. Winder, misunderstands the position taken in the paper. In regard to what he says about the Medical Colleges turning the cold shoulder when applied to for aid in the interest of Dentistry,

I think it was proper they should do so, for comparatively nothing was known of it, at least not enough to call it a science. Dentistry *is* a branch of Medicine, and should be so considered, and my object is to make it essential that a dentist shall receive the degree of M. D., before taking that of D. D. S. We are not asking to be recognized by the medical fraternity because we are members of that profession, but we wish to become members and then demand the recognition. Our professions are so closely allied that they must ultimately come together ; and there is no denying the fact that we can never secure the respect of the world until we do come together. I believe I am right in this, and that I shall live to see the time when it will be an honor to have suggested it. We have made more progress in our profession in the South during the past fifteen or twenty years than any profession in any section ever has done in the same time. Our motto is progress; and in order to secure and keep up this progress we should take a bold stand in this matter, and fight it out.

Prof. Winder.—I perfectly understand the position, but would ask ; why is Dentistry a specialty of Medicine? Simply because certain branches of knowledge are essential to both professions. Surely no one would say that Astronomy was a specialty of Mathematics, and yet it requires a thorough knowledge of Mathematics to undsrstand it. I repeat it, Dentistry is as distinct from Medicine as it is from mechanics, and few would claim it to be a branch of mechanics on account of the mechanical work in its practice. Medicine is so progressive, is covering so large a field, that no M. D. can keep fully up with his profession, and I am opposed to trusting an already over burdened science with the care of our noble profession.

This matter requires mature thought and deep study. I myself don't feel competent to take any step which seems unwise and dangerous, unless you can see the ultimate end. I have no desire except for the best interests of our profession.

Dr. R. Finley Hunt, D. C.—I agree with Dr. Winkler in the principles expressed in his paper, but not with the conclusions he draws. This is no new thing, for in 1875 an effort was made to merge Dentistry into M-D-ism as Dr. Winder has rightly expressed it. We want to keep Dentistry and Medicine as distinct and separate as they now are.

At the last meeting of the National Medical Association, held in Richmond, several dentists from Illinois attended the meeting, and succeeded in obtaining seats, on the ground that Dentistry was a specialty of Medicine as represented by M-D-ism. I do not grant that they had any right or claim to make Dentistry subsidiary in this way to Medicine.

If we as a profession, advance steadily in the same line of march pursued for the last fifty years, and still do not make our profession a specialty of Medicine, the fact that it is so practically will be recognized by the world, and Dentistry will take the position among sciences to which it is rightly entitled.

Dr. Winkler starts out well. I agree with the principles laid down in his paper, but I cannot go with him as far as his verbal remarks carry him.

I believe it would be an advantage to enlarge the curriculum of our Dental Colleges and embrace all of that part of Medicine that may be necessary or useful in our specialty.

I think we are not only even with Medicine, and I say it with all respect to the Physicians and full appreciation of difference, but ahead of it. Dentistry is much more certain than Medicine, and necessarily so, for the Physician has many more complications to deal with than we

Dr. Winkler.—We were asked just now if we would call Astronomy a specialty of Mathematics. No, of course not ; but I should like to know what would Astronomy be without Mathematics ? Dr. Hunt says he is not willing that Medicine should take us in as a specialty. I will ask from whose libraries do we get the information that makes us scientific men? Are we not indebted to Medicine for some of the most important points in Dentistry? I want to magnify and enlarge this grand old profession by every means in our power, and we have the power now to do this of our own accord, which is surely better than to wait and be swallowed up, as we will be eventually.

Dr. Ford, of Georgia.—I want to make all progress in our calling; I am willing to join hands with the M. D's at any time, but I think the overtures should come from the other side. Let us improve our Dental Schools as much as possible, if necessary establish a chair of Medicine in them, but as we are independent now by all means let us remain so. I will accept an invitation from them, but I won't invite myself to any man's house. Let them invite us respectfully, and we will go as equals—as first-class passengers, not as freight or ballast in the old ship of Medicine. For my part, I am 'too proud to beg and too honest to steal.' Let us go on by ourselves, and say to Medicine, 'Come on, we are going to the same harbor, and are willing to help you all we can.' We are able to travel alone and don't want to hang on to anybody's coat tail. As for using their text-books, are they not published to the world? Yes, Mr. President, while I have the most friendly feeling, for them I am not willing to go as a steward or take a low seat in the synagogue.

Prof. Hodgkin spoke briefly in opposition to the leading idea of the paper, and stated that it was no time for dentists to be toadying to M. D's when the latter were coming to us for advice about their own practice, citing cases.

Dr. J. H. Walker opposed the paper, agreeing with Dr. Ford; as did Drs. Hoppes, and Ross of South Carolina.

Dr. Hunt.—I would like to ask Dr. Winkler if he advocates making Dentistry a specialty of Medicine.

Dr. Winkler read the section of his paper referring to preliminary education of Dentists. Of course the general M. D. is not qualified to practice Dentistry; does not know as much on that subject as we do, nor does he know as much about the eye as the oculist. But the main idea I wish to inculcate is that every dentist should be a thorough M. D. before he becomes a D. D. S.

Dr. Hunt.—The fact that a man is an M. D., is no objection to his becoming a D. D. S. But I don't see that he would make a better dentist from that fact than one who was qualified to take the degree of D. D. S. Compare the graduates of Dental and Medical Schools, and it will be found that a Dental graduate is much better prepared to practice general Medicine than the M. D. to practice Dentistry. This fact shows the absurdity of making Dentistry a specialty of Medicine or requiring the degree of M. D., in Dental Education.

Dr. Winkler.—I see I am all alone, but as I feel certain I am right I will not give up to force of numbers. Dentistry may possibly be divided into three classes, not more, while Medicine already embraces a large number of specialties and the number is increasing every day. I know I shall not be able to convince you all, but I know I am right.

Dr. Walker, of Louisiana.—If this were a good thing to do, the present is a bad time for it, as the Medical Profession is already covering too large a field, and must in the near future break up into specialties. Wait till the break comes and then take the lead.

Dr. H. M. Grant, of Virginia.—I came to listen and not to talk, but I must say a word on this subject. I agree with Dr. Winkler, and endorse every word in that paper. I am fortunate or unfortunate enough to have taken the degree of M. D. before that of D. D. S. Not that it makes me more proficient in Dentistry than others, still I think I am benefited by it. I say plainly that Dentistry is simply a specialty of Medicine, and I mean what I say. The field of Medicine is becoming so huge that life is too short for any one man to learn it all. Dentistry is a specialty of Medicine, as it treats of the diseases of the oral cavity alone, while Medicine treats of the whole human system. I believe the day is not far distant when we shall be invited to the feast, and we should have the wedding garment ready.

Dr. Winder.—I think Dr. Grant goes too far when he says we are a specialty of Medicine, unless he means by that term to embrace the whole healing art. In Austin Flint's report to the Medical Convention at New York, in 1876, he calls attention to the progress made in Medicine during the past hundred years, mentioning among other things O. W. Holmes' eulogy on anæsthetics, and in the same sentence regrets that so great a discovery should have come from such a source. In the same paper he says that pulling a tooth is not a surgical operation at all. The truth is, there are some men in our profession (I am not referring to my friend Dr. Winkler, for I know he is above such a feeling,) who wants to climb into respectability by hanging on to the coat tails of physicians. Surgery is to-day the highest round in the medical ladder, as it is the only scientific one yet; to have called a man a surgeon one hundred years ago in England, was to have placed him on an equality with barbers and hair-dressers. If we keep as we are and work on in the right direction, Dentistry is bound to come to the top. If we bide our time and do our duty it must come.

Dr. Ford.—I agree with Dr. Winder, that if we do our duty Dentistry will take her place along side of Surgery.

Dr. Chisholm, of Alabama.—This subject has been a Killkenny cat-fight at every meeting for the last fifteen or twenty years; while in some particulars it may be unfortunate, in others it is beneficial. In fact we do owe something to Medicine, but we are not a specialty but a distinct profession, and I hope we will keep up the distinction, and the integrity of our institution. The two professions respect each other more every day, and the tendency is to give to each his due. The important point in this ques- is the preparatory education of dentists. This is in some cases very deficient, and should be remembered.

Dr. Winder.—I have got this thing to fight in my State Legislature. We want to get a dental law passed, and some, principally country doctors, contend that the title of M. D. entitles a man to do whatever he will with the human form.

The following paper was read by Prof. Hodgkin :

That I am interested in the subject of Dental Education and Literature may be inferred from my presence here. That *you* are interested is proven by your gathering together from village and country cross roads, from the busy city, from home and business.

If I can show some of the lights in which we, whom circumstances have called to be teachers in this matter of education, if I can make plain to your hearts and minds some of the difficulties in the way of our giving a young man of the average development such a dental education as will reflect credit on us as teachers, on you as preceptors, and on the graduate himself as a worker, I shall be glad ; for in convincing *you* I may save my co-laborers and myself mortification and disheartening toil.

Far be it from me to discourage a seeker after knowledge ; far be it from me to say to any one who feels either a *call* or an *impulse*—and I beg you to study the difference between a call and an impulse—to study Dentistry. It is not the noblest view to take of our noble calling, to study it because it is an easy way of getting a living—that there are higher views to take of the matter I am sure you will recognize—but even in this mercenary view of it, one may make a good conscientious dentist. But to discourage a boy who feels that he not only has Dentistry in his heart but in his fingers, wrists, eyes,—who is a *born dentist*, to such an one I open my arms and say welcome to our ranks. What I wish to say however to you who send him ; what I am deeply anxious to impress on you who god-father of him is—Dentistry has grown to be a science, deeply and profoundly studied by some, dipped and dabbled in by more, a sealed book I fear to sadly too many, known as yet in its hight and length and depth and breadth and fullness by *none.* We, the necessities of whose positions compel us, if we are honest, to tax our mental resources to the utmost, who are ever pursuing and struggling and trying to learn, feel deeply and profoundly the magnitude of all this, and the littleness of our own attainments. So much humiliated do I myself feel in the presence of my own ignorance, as to tremble and pause and sadden, knowing I can never know even what I feel is of vital importance to be known.

And this science, as you heard so eloquently yesterday, which goes away back into causation ; which extends its inquiries into that mysterious and dark realm of ante-natal life ; which shows the cumulative results of heredity, which essays to grasp the causes which underlie the pathology on which all our work is founded, and which makes it a necessity : into this science and into its study young men are pushed, the young men who are soon to take our places and do our work, so much better as I humbly and firmly trust. And more than this, they are to be as cultured of hand and as dexterous of wrist as we ; for to be simple "scientists" will be to be failures ; Dentistry means *work.*

Here is the essence of my quarrel and contention with you, my brethren of the profession, to-day. *You send us unprepared men.* If you knew how we long for students, of the thrill which stirs the heart of the professor to see rows of upturned, eager faces, receptive minds, willing hearts ; if you knew how it gladdened us to get students. But oh ! if you know how *our* hearts sink when we analyze this class and are dismayed to find that one or two or three only have college training ; that comparatively few have good common school education ; and that to the mass of these who sit before us, we are compelled to adjust ourselves : how for the sake of these poor fellows whose knowledge of even spelling and reading and writing is so limited, we are obliged to simplify and simplify and simplify, until simplification is wearisome to teacher and advanced student; if you knew how we are compelled to avoid technical phrases, and analyze the Greek and make English of the Latin, and do in a dental school the work of primary education; and in addition to all this train the hands and eyes, and lift the hearts of these boys above any sordid principles they may have inborn in them,—I say if you knew all this and felt all this as we who teach feel it, you at least would pity us.

Brethren, can you not save us some of this toil? We want your students; we feel that their natural home should be with us. We promise, and try to realize what we promise, that we will do all we can to make conscientious Dentists of those who come to us. But if you can in any way save us from this up-hill work of primary teaching, you will confer a favor upon us of no small magnitude. Encourage your students to be students indeed. Try and show up to them the high path before them, explain to them the *altitude* of Dentistry; of the importance for their own sakes of being educated men, of the satisfaction of being able to meet the physician on his own ground and discuss pathology with him, and of making him *look up to us.* Show your student the desirableness of knowledge for its own sake, for its own sweet fellowship. Once set him afire thus, and then send him to us, and you will gladden our hearts and give us a new impulse, and as the fortune of our lives has called us to be teachers, we too will work to see that as leaders we will climb to higher hights yet, inciting in the student's mind such a thirst for a knowledge of the *ultimates* of Dentistry as shall make our profession more than respectable, reputable, honorable, in the highest degree glorious.

My heart is in this work, as those who know me know full well. My ambition is for my calling. I feel it ennobles me; I realize that its study makes me better. I cannot always teach; some of you who hear me may. But as the future of Dentistry is our future, let us see to it that no act of ours hinders its upward progress, that no student of ours is a dead weight on its growth.

Brethren of the Southern Dental Association, I beg you to realize that while the colleges are the exponents and illustrations of the progress of our Profession, and that the teachers realize that they are, whether they will it or not, looked up to as *leaders*, that still *these colleges are what the Profession make them.* Demand of us a high grade of teaching (and you do this by sending us cultured men as students) and we are *obliged* to respond; degrade us by sending us the ignorant and uncultured, and you, if you do not drag us down personally, at least lower the tone of our teaching and so degrade the work we are elected to do. We do want our three hundred seats filled; we should be less then men if we did not; but we do so desire and long for three hundred cultured men, who, already full of general, are ready for special, training. Then shall the millennium of Dentistry come, and dental teaching and dental colleges come up to our ideal.

Dr. Grant, of Virginia.—I wish simply to say that I endorse that paper of Prof. Hodgkin's heartily. It is my position exactly.

Prof. Winder.—I desire to offer some resolutions placing this Association on record as endorsing the position of the Johns Hopkins University of Baltimore, and the University of Virginia, in granting diplomas whenever deserved. A diploma should be simply a certificate of certain attainments, not that its holder has spent so many years in a college. Everything is progression in this age, and the progression which is just in this matter is to ask the candidiate for graduation, not where have you studied and how long, but "what do you know?"

Dr. Ford endorsed the views of Prof. Winder at some length and stated that he wished his son to study dentistry first and then medicine.

Adjourned.

---

*Afternoon Session.*

Secretary Chisholm read several letters from absent members regretting their inability to attend.

The application of Dr. Ed. H. Browne, of Rock Hill, New Jersey, for honorary membership was laid on the table.

The Executive Committee reported favorably on the application of John S. Thomson, D. D. S., of Atlanta, Ga., for active membership, and he was elected.

The subject of Dental Education was resumed.

Dr. Winkler.—I think that the brightest future for Dentistry lies in its becoming a specialty of Medicine. This may be done by, first getting Medical Colleges to introduce Chairs of Dentistry, or second, enlarge our Dental Schools and take in Medicine.

Drs. Hunt and Hodgkin briefly opposed the arguments of Dr. Winkler, and Prof. Hodgkin then introduced the following resolutions, which were unanimously adopted:

"*Resolved,* That the Southern Dental Association place itself on record as endorsing the principles of education as enunciated by the University of Virginia at its foundation, and of the Johns Hopkins University, of Baltimore, Maryland, of conferring degrees according to merit and previous acquirements; and that we endorse heartily the doctrine that a diploma from a dental college should be awarded to merit, and merit alone, irrespective of the number of years of previous study; and, further, that inasmuch as knowledge is equally valuable whenever and wherever obtained, this association indorses, *first,* the doctrine of demanding the highest standard of attainments compatible with the present onward march of dentistry, and on the part of applicants for a degree; and *second,* the most liberal spirit on the part of those granting diplomas as to the best manner in which the said knowledge is obtained; that it is unjust and irrational on the part of any teacher to demand of a pupil the attendance on any prescribed and lengthy course of lectures, if it be proved satisfactorily that the requisite knowledge has been already attained; and that we believe, that in justice to a student of dentistry, as well as of any other study, the privilege of graduating *whenever pronounced fit,* is the true principle.

"*Resolved,* That as a means of facilitating the preparatory work of the student of dentistry, we recommend that no dentist shall take a student for less than two years, and that the failure on the part of such student to possess a good school education should be considered as a grave disqualification for the study of our profession."

The subject of Dental Education was passed and Prof. Winder read the following paper on Microscopy, prefacing it with the remark that he had only a little before been notified of his place on the committee, and that he had received no response from letters to the other members; and further that his studies had not been in that department:

The Microscope is so old that no one is claimed as its discoverer, and its early history is obscure.

The oldest magnifying lens known was found by Dayard in the palace of Nimrod; but whether it was used for microscopical purposes is very questionable and we have no evidence in the case. It is made from rock crystal and is far from being perfect. Lerieca speaks of the magnifying power of a glass globe filled with water. His testimony would date back to the first century, 1800 years ago. But we have no proof that these lenses were used for microscopical purposes in scientific investigations earlier than the 17th century.

We have two kinds of microscopes—simple and compound. The first is composed of a lens or lenses as the case may be, through which the object to be examined is seen directly—in other words the object is only enlarged once. In the compound microscope we have the object first enlarged through a lens or lenses known as the "objective" glass; and there is in addition an eye piece known as the "ocular" glass, which again enlarges the image seen through the "objective." Remove the "ocular" and you have the "simple" microscope, add the "ocular" and you have the "compound," which is decidedly of more value and is now used almost exclusively by microscopists.

The earliest investigations of Marlpighi, Libherkuhn and others, prove that even the simple microscope was a powerful auxiliary in the study of minute objects. I do not deem it necessary to go further into its history or description in this paper. Like every other instrument the hand of progress has constantly sought to over-come difficulties and obstacles and to so improve it generally as to make it better suit the demand of the times in scientific investigation. In this work it has become a most valuable adjunct, reducing the tissues of the body both in the animal and vegetable worlds to their proper anatomical elements, physiologically considered, and enabling the scientist to detect accurately and with positive certainty the aberations from there normal type of structure, thus laying a bottom rock of indisputable facts, upon which to rest our knowledge and study of Pathology. In this way the microscope has led our footsteps through the benighted pathway of Histology as a beacon-light. It is now aiding us as an instrument of diagnosis and prognosis, and is lending its influence to lead Medicine out of the pits of ignorance into the field of knowledge, in order that it may become more thorough, more certain and more scientific.

It has revealed to the vision of man the structure and surroundings of protoplasm—and to the vital strings (discovered by Carl Heitzman, of New York) which bind I might say, life to life in the process of "Histolysis" and "Histogenesis." It has spoken authoritatively in regard to the ebb and flow of the aurate tide which nourishes and cherishes these labyrinths of gossamer-like structures which go to constitute the very existence of life itself—which in the terse language of Bichat has been defined "organization in action." It has invaded the world of fauna and flora, both in the present and past, and enables us to better investigate and understand how to classify and study them. It has revealed the intimate morphological structure of things animate and inanimate. Fossil remains are made to speak intelligently, and the processes of organic development are laid open and exposed. It has filled up the imaginary gulf which was supposed to exist between the animal and vegetable kingdoms, and its latest testimony is convincing to my mind at least, that no such line of separation exists. We are taught more and more every day that there are great rural laws governing the laws of development, structure, form, and even life itself. In the language of Dr. James L. Calleb, of the University of Virginia, there is a "unity of force amongst a diversity of phenomena." The changes brought about, not by any radical alteration of the general law itself, but surrounding forces or "environment," have so influenced the result of the general law in individual cases, that constant modifications of the rudimentary form are exhibited and endless variety meets us at every step. But it is the "unity of force," modified, not destroyed, that presents the ever varying kaleidescope of the "diversity of phenomena."

Let us for a moment take a retrospective view—when microscopy had thrown no light upon these matters.

In drawing the line between the animal and vegetable kingdoms, it was decided that it all depended upon a stomach. Its presence demonstrated the fact of animal life; its absence proved the presence of vegetable life. This theory was satisfactory for a time, but later, plants were discovered with *bona fide* stomachs, whose digestive functions were essential to the comfort and welfare of the plant, so that this line of demarkation and separation had to be changed in accordance with the requirements of the nearly known facts.

Then came the theory that "sensation" and "voluntary motion," (a theory still adhered to by some of our leading teachers) formed the dividing line; that when these two phenomena were exhibited, that there animal life must be present. For many years, while this theory was being taught from our best text-books, there were thinking minds who doubted its truth. The action of the sensitive plant was too nearly akin to the sense of feeling with its reflex nervous action (in closing its leaves) to accurately discriminate between "sensation" and "sensitiveness," the two terms selected to represent in the animal and vegetable worlds respectively, what, it would in all probability, be proper to ascribe in both to the one law of "unity" or

"nervous influence." Then again, the question of "voluntary movement," as a dividing line, where investigated, seemed too much like a dissolving view to be relied upon.

A plant placed in the dark, which requires light, would always and by some inevitable law seek any aperture that could shed upon it a ray of light; the habit of running vines to twist in one direction show that the power of movement seems at all events to dwell in the plant itself; that it is an automatic movement as much so as the movements of the heart. This course of analysis and reasoning carried its own logical deductions, and we had therefore our skeptics (and I confess I was one of them) who doubted the validity of any such line of separation as was marked out by the prescribed terms of "Sensation" and "Voluntary Motion," and hence the long trial of the question whether sponges were vegetable or animal organisms. We have also until very recently been taught that while animals in respiration gave off carbonic acid gas and took in oxygen, that in the vegetable world the reverse was the case, and this was taught most dogmatically. We know better now, and much of this enlightenment is due to the valuable aid of the microscope. As the minute structure of tissue was revealed, it naturally suggested its physiological functions.

The latest revelations of the microscope, so recent indeed, that Prof. Foster's work is the only text-book in Physiology which treats of these matters, seems to settle forever the questionable line between the animal and vegetable kingdoms, filling entirely the gulf which was supposed to exist and totally effacing the last lingering doubt.

Prof. Foster says, "among the simpler organisms known to Biology, perhaps the most simple as well as most common is that which has received the name of Amœba." These are common to both the animal and vegetable kingdoms and possess the same general characteristics. They closely resemble the white corpuscles of vertebræ blood.

They are wholly or almost wholly composed of undifferentiated protoplasm, in the midst of which lies a nucleus—though this is sometimes absent. In many a distinction may be observed between a more solid external layer the ectosarc, and a more fluid granular interior or endosarc—but in others even this primary differentiation is wanting. By means of a continually recurring flex of its protoplasmic substance the amœba is enabled from moment to moment, not only to change its form, but also to shift its position. By flowing around the substances which it meets in a manner, it swallows them and having digested and absorbed such parts as are suitable for food, ejects, or rather flows away from the useless remnants. It thus lives, moves, eats, grows, and after a time dies, having been, during its whole life, no more than a minute lump of protoplasm. Hence to the Physiologist it is of the greatest interest, since, in its life, the problems of physiology are reduced to their simplest forms. Now the study of an Amœba, with the help of the knowledge gained by the examination of more complex bodies, enables us to state that the undifferentiated protoplasm of which its body is so largely composed, possesses certain fundamental, vital properties.

1st. It is contractile—representing muscle force.

2d. " " irritable and automatic—nervous action.

3d. " " receptive and assimilative—digestive action.

4th. " " metabolic and assimilative—secretive and ex cretive.

5th. " " respiratory—giving off carbonic acid and taking in oxygen.

6th. It is reproductive.

So we have here the evidence that one general law pervades organic life, from the simplest to the most complex. "A unity of force amongst a diversity of phenomena."

It would be a pleasant pastime to follow this subject further and discuss it from a physiological standpoint, but this paper is supposed to be confined to another matter.

All these secrets from nature have been brought to man's understanding through the medium of Microscopy, and these revelations have settled in my mind the question of a dividing line between the animal and vegetable kingdoms. I believe no such line exists; possibly at some future day further revelations may throw a doubt upon the fact of a dividing line even between the organic and inorganic worlds. So far as we now understand the matter, the same general laws apply here. To construct there must be building material, and there must also be some power by which this material can be wrought into structure, and this fact obtains equally in the organic and inorganic worlds. In the latter, we have material and there is some canny power which converts that material into the beautiful crystalline forms which are exhibited in the field of the microscope, each *sui generis* and distinctive in its structure. Surely we have here too, a "unity of force amongst a diversity of phenomena."

I have called your attention now to the great value and importance of the microscope as an auxiliary to scientific investigations generally. I propose at some future day to write two more papers. One will be the Miscroscope and its Revelations in Physiology, and the other will be, the Microscope and its Revelations in Pathology.

Before finishing this paper however, I would impress upon my hearers, what I try to impress upon every dental student that comes under my influence. That absolute necessity requires a dentist to take the best care possible of his eyes and hands, and therefore I earnestly advise them to let others do the work in Microscopy and reap the benefit of their labor. The eyes of a dentist are used to excess in his daily routine of fine operative work in the mouth; to further tax them at night with a microscope, would be simply ruinous to them. Of course, if they must see these things for themselves—and it takes long years of practice to see them intelligently—the binocular microscope should be used, as the strain in the use of this instrument is equally divided between the eyes, and consequently not so injurious.

The subject of Microscopy was then passed, and Dental Chemistry called.

Dr. Winkler stated his belief that an alkalin was the cause of decay under the margins of the gums, and thought he had proved this.

Dr. Chisholm thought it was an acid.

Dr. Hunt gave it as his belief, founded on many observations, that it was an alkali which caused the decay in question. The remedy, among others said to be useful is nitrate of silver.

Dr. Griffith hesitated to discuss Chemistry and related a humorous story connected with his final examination of the professor of Chemistry when at college.

Dr. Hunt in response to a question from Dr. Carr, stated that the smoothly polished groove sometimes on the neck of the tooth was caused by a chemical agent and not by the brush as was commonly thought. How it was so highly polished he could not answer. The chemical reaction of the mucous secretion in such cases is always alkaline.

Adjourned.

---

*Night Session.*

The following paper on Caries of the Teeth, by Dr. J. D. Clark, of Newbern, North Carolina, was read:

Caries of the teeth, although a misnomer, is the disintegrating or breaking down of the structure of the teeth, a disease well known to every dentist; and yet how little it is understood is shown in every effort to stop its ravages, or to prevent its occurrence, at times proving futile.

It is admitted that dental caries results from chemical action. There are constitutional causes, producing badly organized, defective teeth; an l in originating destructive agents, which act on them.

We must bear in mind that the teeth are living organs, and physiologically not subject to spontaneous decomposition or disintegration; and to understand their pathological deterioration we must, first, knowing the composition of the teeth, learn the character of the destructive agent or agents.

Analyses of teeth vary accordingly as the teeth analyzed are well or poorly calcified; or are the teeth of old, or young subjects. It will suffice to say, that the enamel is composed of about four parts of organic, and ninety-six parts of inorganic matter; and the dentine, of twenty-five parts of the former, and seventy-five parts of the latter. The inorganic portion of the teeth is composed principally of phosphate of lime, with carbonate of lime, magnesia, sodium chloride, etc., and the organic, like other parts of the body, is made up of albumen, casein and fibrin, which are subject to the same chemical reactions here, as elsewhere in the system.

We would naturally infer from the composition of the teeth, that the deleterious agent is an acid. Caries almost invariably attacks first the approximal and buccal surfaces, and the sulci of the teeth, because they are least exposed to friction; and it is at these points that food is allowed to remain and decompose, thereby producing acids, which corrode them.

Please bear in mind that chemical action is always definite. No two acids can unite with the same base, to produce the same compound. In other words, the compound, or salt, formed by an acid with a base, or alkali, is distinguished by chemical characteristics peculiar to itself, from all other salts. The compounds thus produced, are always definite, because they are formed in obedience to established laws.

As it is admitted that dental caries is the result of the chemical action of acid on the teeth, we are naturally led to ask, Is caries the result of one acid, or more than one? Are there more varieties than one, of dental caries?

Let us notice some of the characteristics presented by this disintegrating process known as caries.

I know that you are well aware that the phenomena presented by caries are few in number, and that the profession, as if by common consent, recognize only three kinds; and if we include chemical abrasion, then four kinds.

The "white decay" so familiar to us and so destructive in its work, advances more rapidly than the other varieties. The chemical reagent at work here not only acts on the inorganic material of the teeth, but, also, on the organic. In the second or "brown" variety, the inorganic matter seems to be acted on only, and the organic substance remains behind as a coating to the cavity; and in the third kind, or "black decay," the destructive agent acts less rapidly than in either of the other varieties, seeming to form insoluble compounds with both the mineral and animal matter. When chemical abrasion attacks the teeth, they are entirely destroyed as far as the disease extends; and it appears, that the agent at work forms soluble compounds with the organic, as well as the inorganic matter.

Now since the phenomena of caries are few, and chemical compounds are always definite, must not the inference be that the reagents, which produce dental caries, are also few? If only one acid produces the different kinds of caries, or if two or more acids can unite with the same base, to form salts of the same chemical composition, attended by the same chemical phenomena, then compounds are not definite; but since it is a law of chemistry that compounds are definite, we have no other conclusion than that stated above.

It is very probable that each variety of caries is produced by a single agent.

The action of acids on the teeth depends on the degree of their concentration, and, also, on the physical characteristics of the teeth. When much diluted, an acid unites with that substance for which it has the strongest affinity. The softer the tooth, the more rapid is its action, as the acid is brought into more immediate contact with the component parts of the tooth, than when its structure is dense.

Where are we to look for the acids, which produce such disastrous results, so distinct from each other in their chemical characteristics? Surely not among acid medicines, nor acid food, though they may, and do, at times, seriously injure the teeth. They act as predisposing causes by roughening and pitting the teeth, which would otherwise remain smooth and polished, a condition necessary for their preservation. The reagent must be sought for in the secretions or excretions of the mouth, which are prepared and eliminated by the system; and in the products of the decomposition of food, allowed to remain about and between the teeth, through uncleanliness or neglect.

The limits of this paper will not permit a minute consideration of the secretions of the mouth, yet not to pass them over entirely, let us hurriedly glance at their composition, and the probable part which they play in the destruction of the teeth.

Normal saliva is a limpid fluid, with slight degree of viscidity, and has an alkaline reaction. It is composed of water, and a small quantity of solid matter, as albumen, fat, ptyalin, sulpho-cyanogen, and spirit and water extracts.

The salts usually found in the saliva are phosphates, lactates, carbonates, chlorides, and traces of sulphates.

Abnormal saliva has an acid reaction; and the acids usually present are lactic, acetic, hydrochloric, oxalic, uric, &c.

Mucous is obtained in such small quantity, and is so variable in consequence of any irritation of the membranes, that it is difficult to characterize. However, we know that it contains extractive matter, alkaline lactates, chlorides of sodium and potassium, and a small quantity of fat. It also contains a peculiar nitrogenous principle called mucin.

There is a secretion of the gums, about which but little is known. Some attributing to it an alkaline reaction, while others state positively that it is decidedly acid.

The saliva, as it is found in the mouth, is a mixture of the above secretions; and, that it is seldom found to be normal or alkaline, I think very probable. In a small number of tests of the saliva, or mixed secretions of the mouth, of about one or two hundred children and several adults, the reaction in nearly every instance, was decidedly acid.

Let us now briefly consider one or two acids, which do act on the teeth, producing results similar to those of caries.

Nitric acid, which is composed of one equivalent of nitrogen united with five of oxygen, acts with energy on the teeth. It dissolves the phosphate of lime, decomposes the carbonate, thus setting free carbonic acid forming nitrate of lime. The organic portion is entirely destroyed, and a very softened condition is produced, resembling "white decay." It is very likely that this acid is the active agent in the production of this variety of caries. Nitric acid unites readily with the bases; and in the presence of weaker acids, it takes these bases from them.

Although nitrogen and oxygen manifest but little affinity for each other, they unite to form five definite compounds. We are told that their tendency is to unite in the proportions to form nitric acid; and all that is necessary for these changes is air or moisture. So that, when one of these compounds of nitrogen and oxygen is formed, it is eventually converted into nitric acid by the oxygen of the water or air, both of which are always present in the mouth.

We must not be led to suppose that nitric acid is the result of the union of the oxygen of the air, or water, with the nitrogen which is liberated by the decomposition of the mucus, and particles of nitrogenous food, which are allowed to remain about and between the teeth. Hydrogen is always present, as well as nitrogen and oxygen, in the decomposition of organized nitrogenous substances. The mutual affinities of hydrogen and nitrogen take precedence, thus forming ammonia. Now ammonia, when exposed to oxygen, decomposes; and an oxide of nitrogen is formed, which is finally converted into nitric acid.

The second, and only other acid which I will mention is sulphuric.

I know that it is claimed by many that this acid does not injure the teeth, even when they are frequently bathed in a strong solution of it. Why it does not, has never been explained; and it seems strange, that an acid like this one, which has a strong affinity for alkaline bases, when brought in contact with the teeth, should remain inactive. It has been proven, by recent experiments, that sulphuric acid does not act on living bone. The teeth have a very similar composition to that of bone, differing from it in containing less organic matter, and in being denser and firmer. Then why does it not affect the teeth?

Sulphuric acid, having a strong affinity for alkaline bases, does not act directly on the phosphate of lime in the teeth, because it is not the neutral, but a sub-phosphate. It is neither soluble in this acid, nor is it decomposed by it; but the other simple inorganic ingredients of the teeth are, and by their slow removal the teeth are broken down.

The affinity of sulphuric acid for water is so energetic, that it often forces its elements to forsake favorite combinations, to unite with it. In this way carbonization of the organic constituents of the teeth is produced.

If this acid is formed in the mouth, how is it formed there?

Sulphur, when it unites with oxygen, has a tendency to form sulphuric acid. But is sulphur present in the mouth? We know that it is united with albumen, and that albumen is a constituent of mucous, and is found in many kinds of food. It is probable, however, that it originates in an indirect manner. Hydrosulphuric acid, or sulphuretted hydrogen, is a product of the decomposition of albuminous substances. That it is found in the mouth, and is exceedingly offensive, I know you will agree with me. The oxygen of the air decomposes this acid by uniting with its hydrogen to form water. The sulphur thus liberated, being in the nacent state, has its affinities increased in energy, and unites with oxygen to form sulphurous acid, which, in presence of the water of the saliva, is converted into sulphuric acid.

Having seen that the action of this acid is slow, and that it produces carbonization of the organic portion of the teeth, phenomena which characterize "black decay," shall we deny it all power in the production of this variety of caries?

In conclusion, let us sum up what has been said:

1st. Caries of the teeth is due to the chemical action of acids.

2nd. Chemical action is always definite.

3rd. The teeth are composed principally of alkaline bases.

4th. Acids, which have a strong affinity for these alkaline bases, are found in the saliva.

5th. The compounds, foamed by acids with bases are always definite.

6th. The varieties of caries are few.

7th. The chemical characteristics of caries are similar to the results produced by the action of certain acids, which are contained in the saliva.

There are many things which should have been noticed in considering a subject like this, but time would not permit it.

Dr. Hunt.—I must say a few words in commendation of this paper and others by young men. It indicates that the young are preparing themselves to take the places of the old men as they drop out, and I am glad to see it. I cannot agree however with Dr. Clark in thinking that acid is the only agent in the destruction of teeth.

Prof. Hodgkin and Dr. Winkler also spoke in warm terms of admiration of the essay of Dr. Clark, the latter stating that he gathered from the paper reasons for clinging to his alkaline theory as causing decay in some cases, though acid was doubtless the principal cause.

Dr. Hunter.—There is one point on which I want light. I have a patient whose teeth are decaying around the gums as described, and yet he complains of an acid taste in the mouth. If alkali is the agent how can the acid taste be accounted for?

Dr. Winkler.—The gum being diseased, instead of secreting the natural fluid, secretes a strong solution of ammonia directly on the teeth, where it has time to act before the acid in the mouth counteracts it.

Dr. Winder.—I commend the paper strongly; it shows careful study. Such papers are valuable in the extreme. The chemistry of the mouth is the only means of solving the problem of the decay of teeth.

Dr. Walker.—There is another important point in the chemistry of the mouth, and that is the origin or cause of tartar on the teeth. It has been my experience that tartar in some extreme cases is caused by eating hot grease or butter. I have often told my patients when they would bring me a bad case of tartar that they must stop eating hot grease, when they expressed surprise as to how I knew it, confessing its truth.

The subject of Dental Chemistry was passed, and Dr. Griffith, on the part of the North Carolina State Dental Association invited the Southern Dental Association to a banquet prepared in their honor, for to-morrow night. The Association accepted the invitation and then adjourned.

---

THIRD DAY,
*Morning Session.*

On motion of Dr. Hunt it was resolved that a committee of three be appointed to revise the roll of the Association. The President announced that he would reserve the appointments for the present.

Resolutions expressive of the regret and indignation of the Southern people as represented here, at the late attempted assassination of the President of the United States, were introduced by Dr. Winkler and adopted unanimously.

The subject of Operative Dentistry was called and temporarily passed, and Dr. Winkler called the attention of the Association to the subject of plastic fillings, to which he had devoted a good deal of attention. He said :

My object for some years past has been to find or make a filling with which an unskillful man could save a tooth as well as the best dentist. Gold is too hard and requires too much skill in the manipulation. My filling which comes near doing what I wish, but still does not quite do it is this : Take one ounce of chloride of zinc, and dissolve in it as much silk fibre as it will take up, and carefully digesting this over a spirit lamp. This forms the liquid to combine with the oxide of zinc. When I first used this I liked it very much, but I find by experience that it is best to use sponge in place of the silk. This filling is almost as easily worked as putty and hardens very quickly; in four or five hours it gives a good grinding surface. I think this is the best plastic filling I have ever used. It is very cheap and can be manufactured in the office, and with it a great many teeth may be saved that are too far gone or too frail to save with gold.

Prof. Hodgkin.—I am very glad that Dr. Winkler failed to discover a filling which an unskillful man can handle as well as a dentist. I am devoutly thankful that he has made no such discovery, and I hope he will give up the quest, for I should be sorry indeed if the day should come that teeth can be filled well and durably by any one, no matter how inexperienced or unskillful.

Dr. Walker coincided with Prof. Hodgkin, but trusted that Dr. Winkler would not give up his search for a perfect plastic filling. His own favorite plastic is oxychloride of zinc, with a coat of melted paraffin.

Dr. Winkler claimed that he was misunderstood. He was looking for a filling which would enable comparatively inexperienced young men to save teeth, at present lost by their being poor manipulators.

Dr. Hoppes, of Georgia, read a paper on "Plastic Fillings."

Dr. Ford.—Of the two fillings oxy-phosphate and oxy-chloride, I much prefer the former, especially for capping pulps. The latter kills pulps every time.

Dr. Hunter.—I prepare oxy-phosphate by putting pure oxide of zinc into a clean crucible and heat to the welding point of cast steel for four hours, when cool add equal parts of enamel spar. For the liquid I use phosphoric acid, c. p., such as we get from the dental depots, and make a saturated solution in water. When I want to use it I mix it up on a piece of glass, and it works very much like putty. I can put it into any shape. This gives me just about time enough to do my work before it hardens. Ten minutes after I put it in the tooth, I am ready to finish it, and in half an hour I polish it with a little silicate of soda and a warm instrument. But I am afraid of this, as I have had successes and failures with all plastics, certainly no special success. The zinc fillings sometimes fail me and sometimes do not. Gutta Percha has also given trouble sometimes. In one case when I used oxy-phosphate of zinc, the capped pulp was dead in one week.

Dr. Ford thought failures due to capping diseased pulps, but confessed he knew of no means of positively determining when a healthy condition exists.

Dr. Walker endorsed the above remarks. He subjected the pulp, in cases of doubt, to a creasote bath, and then used oxy-chloride of zinc. He had been using for some time the St. Louis nerve dressing; and thought it useful.

Dr. Chisholm preferred to deal with healthy pulps, and capped with old wood, creosote and the powder of the diamond cement, and lately had used this last with oil of cloves and over this some good setting plastic.

Dr. Walker related a case of his where exposed pulps were capped, the capping of oxy-chloride of zinc dissolved away by the oral fluids, re-exposing them, and they were again successfully capped, after two years.

Dr. Hunter said that he found, in contravention of a statement by Dr. Walker, that in most cases he could fill roots of teeth better with gold than anything else.

Dr. Walker replied that he spoke advisedly when he said it was not best to attempt filling roots with gold. Such attempts were nearly always failures. He used oxy-chloride of zinc pumping it in with cotton.

Dr. Hunt thought that while root filling was exceedingly difficult, yet it was not at all impossible, as he had seen many such cases where the gold was compactly carried to the apex of the root. Where roots are curved or flattened some plastic is better.

Dr. Ford said the debate had drifted from the important subject, the capping of pulps. He had one consolation in any troubles in that quarter—the tooth was diseased when he got hold of it. He thought success was pretty sure when oxy-phosphate of zinc was used and no diseased condition was present. He used oxy-chloride of zinc for root fillings.

---

*Afternoon Session.*

Dr. Griffith read a paper on Operative Dentistry, asking for light on certain points discussed in it. [Paper not found among proceedings.—EDS. JOURNAL.]

Dr. Ford referred to the subject of pulp-treatment, and said that disease was the ablest of generals, always attacking the weakest point. He thought that much of this trouble with pulps grew out of constitutional causes.

Dr. T. M. Hunter, of North Carolina, read a paper on Gold Foil, of which the following is a synopsis:

In the November number of the *Dental Luminary*, Dr. T. M. Allen, in discoursing on the causes of failure with cohesive gold, says of those who fail: 1st. The failure to shape the cavity properly, making it round at the bottom and without retaining points, thinking the gold will adhere to the tooth. 2d. Failure to secure dryness. 3d. Overheating and thus making the gold harsh and difficult to handle. 4th. Overpressure or over-malleting—a great mistake. 5th. Condensing the gold at any convenient point, and trying to move it after partial condensation, thus loosening the first piece. 6th Using large rolls or slips, handling it in the same way and finding that the gold has balled up under the instrument and become unmanageable. 7th. If small pieces are used, condensing toward the centre instead of outwardly, and finding the pieces breaking off whilst being handled, or shortly afterwards 8th. Using a napkin instead of rubber dam, and possibly touching the already inserted gold with the fingers.

There are objections to the indiscriminate use of cohesive gold foil even by dexterous hands, directed by trained minds, for instance, in approximal cavities of soft teeth in young subjects, where a somewhat vulnerable point, the cervical wall and margin, is to receive the first force of impact when there must necessarily be but little gold between the plugger and tooth substance, thus rendering a more or less pulverized condition of this wall liable. A perfect filling is *impossible* under these conditions; the most skillful fail often, and should he succeed, the extra time, labor and suffering, or at least discomfort, are not warranted by any subsequent condition of things. We have great confidence in the value of cohesive gold in what we conceive to be its proper use, and have employed it for many years with almost all kinds and weights of mallets as well as hand pressure. In the restoration of broken outlines it has opened a field of valuable service which it does not appear could have been done by any other substance. But as this substance is known to have less plasticity than the so-called non-cohesive foil, and as a greater adaptability is one of the principle requisites of a filling material, why, because the cohesive has greater value in some cases, give it place where the non-cohesive foil is clearly indicated? A majority of dental graduates seem to have little knowledge of non-cohesion, though it is said to be taught in the colleges. They seem to have been after something fancy without thinking it possible to do anything really fine with most cohesive foil. We do not think it is generally believed that non-cohesive foil fillings when finished, may be perfect in density, margins and retention of finish, and also even in shape to a moderate degree of contourity, but it is nevertheless a fact. Perhaps there is no dentist who does not frequently have to remove rough, soft and useless fillings into which he could easily thrust a small plugger, to find to his surprise that it was only marginal decay, most of the cavity being perfectly sound, and upon inquiry ascertain that no dentist had touched them for ten or fifteen years. The whole trouble has been that it did not have sufficient density to receive or retain a finish, no cohesive filling at all approaching it.

Sufficient regularity should be given the cervical position of the cavity to issue perfect immovability of the whole mass of gold from beginning to end of introduction, as in the use of cohesive gold; this method contemplates the same solidity from the beginning of the work. If it is not possible in molar and bicuspid cavities to obtain a cervical wall of fair depth, in proportion to size or width of cavity, a gold of moderate cohesiveness had better be worked from retaining points. To illustrate —take an approximal molar cavity of ordinary size, and strong lateral walls and obtain sufficient space by wedging to pass in a thin separating file, then cut down through the grinding surface, trim and level margins everywhere, undercut most on the more accessible lateral wall, adjust a rubber dam and then a good hard wedge which will reach from the neck to a little above the cervical margin. If this wedge is well adjusted it will save some of the labor incident to removing surplus gold on this margin, which is often difficult. If it would take one sheet of number six gold to fill the approximate parts of this cavity, I cut a sheet in four strips, fold them smoothly into ribbons, and roll about two-thirds of one of them into a cylinder, which is to be put side-ways into the accessible undercut and with a broad well serrated foot—point firmly malleted into it, making all the blows in a direction half-way between the plane of lateral and cervical walls. A small cork-screw hand plugger should be used in the left hand to keep absolute steadiness of the gold while it is being condensed More gold from the ribbons should now be used, gradually extending it along the lateral and cervical wall until each of them have been covered, and that on the cervical has touched the other lateral wall. The marks of condensation should now all be facing the uncovered wall, forming an independent cavity of the remaining unfilled space. The work so far should be as solid as if done with cohesive foil. A suitable small point should now be used to fill the V shaped space. For this purpose one of the ribbons should be cut into small sections, and driven directly into the bottom of the V, and when the cavity is nearly full resort to number six cohesive gold foil the fissure on grinding surface, and building lastly the angle with number sixty or one hundred and twenty rolled gold. When there are walls intact and strong there is no need of cohesive foil. In finishing these fillings a file should not be used, unless it be in the form of a very thin saw to get a start. Preferedly we use very sharp knives, chisels and emery strips. When soft foil can be firmly held by opposing walls and is so built in that each fold carries its unbroken strips in and out the cavity, if it is properly condensed from the start it will finish as beautifully as cohesive gold, and if no extra wear is to come upon it, it will hold the finish as long. Used in the way described it has an easier adaptation to tooth substance, and can be made equally if not more perfect in less time than cohesive foil.

We never use cohesive foil throughout an entire filling except where prolongation beyond the natural walls is to be left in the finished work or when so much of lateral walls are gone as to necessitate starting from a retaining point; although we use some little of it in nearly all cavities. In the fissures of grinding surfaces and on that surface in the main cavities we employ it nearly altogether.

This method is not new, except perhaps some slight individuality in manipulation, but having seen it so often a success in poor teeth when cohesive work throughout had failed, we feel a desire to proclaim it even though "Filling Teeth" is as threadbare in the journals as it is.

Dr. Hunt thought the question of soft and cohesive foils an open one. Either will make a perfect filling, but require different instruments and different manipulation. He used a small pointed instrument for soft foil, driving it down in the centre of the already inserted gold, and thus wedging solidly.

Dr. Winkler thought that there was a greater tendency

South to use soft foil. He used the same instrument for both styles of work, and claimed to have fair success.

Drs. Chisholm and Hodgkin spoke briefly, advocating soft foil, though not condemning the other.

Dr. Grant used both styles of gold but was not wedded to either. Such means as proved the best results was his motto. He recently saw a filling that had lasted for fifty-one years, and giving no signs of failure yet. When he saw this he felt like crying "Eureka." Careful handling was the main thing.

Drs. Ford and Walker spoke in the same strain.

Dr. Hopps would not give up either and used both in the same cavity.

Dr. Chisholm spoke of a tin filling forty-three years old still in good condition.

Dr. Hape, of the Atlanta, Georgia, Dental Depot, spoke by invitation on gold foil and its manufacture. All fine gold, he said was cohesive. If not so it was due to either surface contamination or alloying, usually with copper. An alloy of silver with gold does not destroy its cohesiveness. Exposure to the air made cohesive foil non-cohesive, but this was regained by annealing.

Adjourned.

---

*Night Session.*

Drs. R. P. Bessant, W. H. Hoffman, and J. T. Calvert, of North Carolina, were elected members of the Association.

The Association then proceeded to elect officers for the ensuing year, with the following result:

President: Dr. E. S. Chisholm, Tuscaloosa, Alabama.
1st. Vice President: Dr. Geo. W. Winkler, Augusta, Georgia.
2d. " " Dr. E. L. Hunter, Enfield, North Carolina.
3d. " " Dr. Isaac Simpson, Winneboro, South Carolina.
Cor. Secretary, Dr. J. P. Holmes, Macon, Georgia.
Rec. " Dr. W. H. Hoffman, Charlotte, North Carolina.
Treasurer; Dr. H. A. Lowrance, Athens, Georgia.

Pending a discussion as to the next place of meeting, of the Association, adjourned to the Swannanoa Hotel where they partook of a sumptous banquet provided by the North Carolina State Dental Association, which was one of the most pleasant features of the Association, and truly a social reunion.

[CONCLUDED IN NOVEMBER NUMBER.]

